# Nasopharyngeal cancer mimicking otitic barotrauma in a resource-challenged center: a case report

**DOI:** 10.1186/1752-1947-5-532

**Published:** 2011-10-31

**Authors:** Adekunle Daniel, Ayotunde James Fasunla

**Affiliations:** 1 Department of Otorhinolaryngology, College of Medicine and University College Hospital, PMB 5116, Queen Elizabeth Road, Ibadan, Oyo-State, Nigeria

## Abstract

**Introduction:**

Nasopharyngeal cancer commonly manifests with cervical lymphadenopathy, recurrent epistaxis and progressive nasal obstruction. Neuro-ophthalmic and otologic manifestations can also occur. Isolated otologic presentations of nasopharyngeal cancer are rare and the diagnosis of nasopharyngeal cancer may not be foremost in the list of differentials.

**Case presentation:**

We present the case of a 29-year-old Nigerian woman with bilateral conductive hearing loss and tinnitus after air travel. There were no other symptoms. The persistence of the symptoms after adequate treatment for otitic barotrauma necessitated re-evaluation, which led to a diagnosis of nasopharyngeal cancer.

**Conclusion:**

Isolated otologic manifestations of nasopharyngeal cancer are rare in regions with low incidence of the disease. There is a need for it to be considered as a possible differential in patients presenting with bilateral serous otitis media.

## Introduction

The clinical presentations of nasopharyngeal cancer may sometimes be insidious and nonspecific. They are usually related to the local, regional and distant spread or metastasis of the lesion. They may include cervical lymphadenopathy, nasal blockage, epistaxis, hyponasal speech and otologic and neuro-ophthalmic manifestations [1]. The clinical morphology of the lesion may be infiltrative, ulcerative or exophytic.

The otological manifestations of this disease entity are commonly unilateral Eustachian tube dysfunction, fluid accumulation within the middle ear, conductive hearing loss, otalgia and tinnitus [2]. However, these presentations are not pathognomonic of nasopharyngeal cancer. It is quite uncommon for nasopharyngeal cancer patients to present with only isolated otologic symptoms, especially in regions where the incidence of this disease is low. When they do occur, other more common benign ear diseases that present with similar symptoms are usually considered. A high index of suspicion is required to evaluate the patient for nasopharyngeal cancer as a differential diagnosis. Hence, we report an unexpected presentation of nasopharyngeal cancer, with isolated otologic symptoms, which was initially managed as otitic barotrauma.

## Case presentation

A 29-year-old Nigerian woman, who frequently travels by air, presented with a six-month history of persistent bilateral hearing impairment following a flight. She erstwhile had experienced repeated episodes of this symptom, which occurred each time she flew, but there was always complete resolution after a few days following treatment from an outside health facility. There was associated tinnitus but no otalgia, no ear discharge and no sensation of disequilibrium or vertiginous spells. She did not have any nasal blockage, nasal discharge, epistaxis or postnasal drip. There were no throat or neuro-ophthalmic symptoms. She did not complain of neck swelling. There was no history suggestive of exposure to carcinogens.

She had received treatment at peripheral hospitals for barotrauma before presenting to our hospital due to persistence of the symptoms.

Examination revealed a young woman with a nevus on the lobule of her right pinna. Both tympanic membranes were dull with a loss of light reflex. The tuning fork test showed evidence of bilateral conductive hearing loss. No evidence of spontaneous nystagmus was noted. A nasal and oropharyngeal examination revealed essentially normal findings. Indirect laryngoscopy findings appeared normal. Her cranial nerves and both eyes were grossly normal. Examination of her other systems did not reveal any abnormalities.

**Figure 1:**
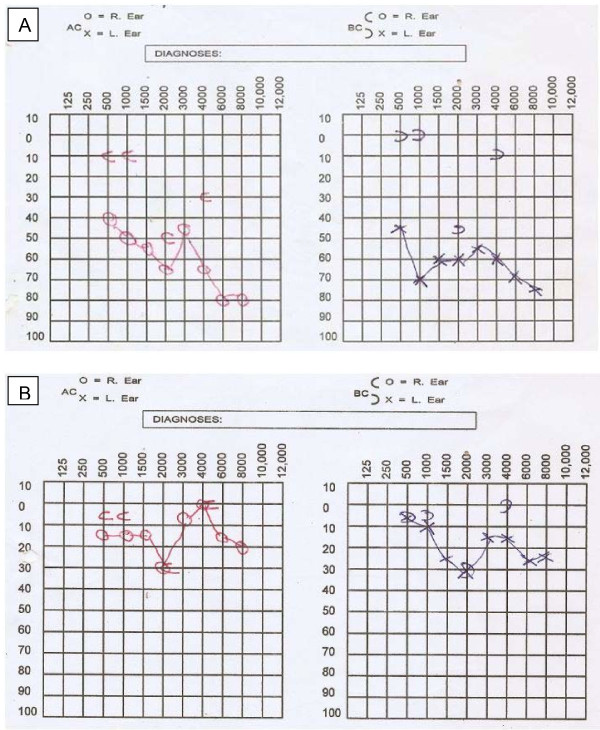
**Pure tone audiogram**. (**A) **Audiogram of our patient at presentation with evidence of bilateral conductive hearing loss. **(B) **Audiogram shows improvement in hearing thresholds after commencement of treatment.

A pure tone audiogram confirmed the bilateral conductive hearing loss (Figure 1A). Impedance audiometry showed type B curves bilaterally.

**Figure 2:**
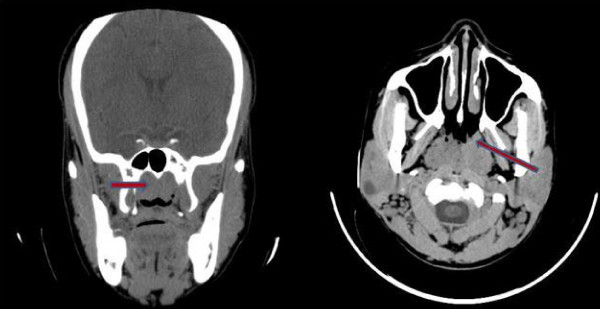
**Computerized tomography scan of our patient shows an isodense lesion in her nasopharynx**.

A diagnosis of bilateral otitic barotrauma was made. She was treated with nasal decongestants, prophylactic antibiotics and asked to perform the Vasalva maneuver frequently. However, her symptoms still persisted after two weeks. This necessitated a re-evaluation; during examination her tympanic membranes were now hyperemic and bulging. A computerized tomographic (CT) scan of her paranasal sinuses was done, which revealed isodense lesions in both fossae of Rosenmüller with complete occlusion of the openings of the Eustachian tubes bilaterally (Figure 2). Nasopharyngoscopy, which would have been pivotal in reaching a diagnosis, was not done before the CT scan because nasopharyngeal cancer had not been in our list of differentials. She underwent examination of the nasopharynx under general anesthesia and a biopsy of the lesion was performed. The histology revealed an undifferentiated carcinoma of the nasopharynx (World Health Organization type III). She was referred to the clinical oncologist and radiotherapist in our center for treatment. The hearing loss improved after commencement of chemoradiation; a pure tone audiogram thereafter showed socially adequate hearing thresholds in most frequencies (Figure 1B).

## Discussion

This present study clearly demonstrates a case of bilateral serous otitis media which was the only clinical finding in a patient who was initially thought to have otitic barotrauma. Thorough evaluation after the failure of initial treatment led to a diagnosis of nasopharyngeal cancer. The otologic manifestations of nasopharyngeal cancer are usually unilateral. Bilateral presentation is quite uncommon [3]. Bilateral serous otitis media or Eustachian tube dysfunction as the only clinical manifestation of nasopharyngeal cancer is uncommon and rarely reported in the literature. A high index of suspicion is therefore needed to evaluate patients with bilateral serous otitis media or Eustachian tube dysfunction for possible nasopharyngeal cancer.

The otologic manifestations of nasopharyngeal cancer occur as a result of the sheer tumor bulk within the nasopharynx and paranasopharyngeal space extension [4,5]. These manifestations may include Eustachian tube dysfunction, fluid accumulation within the middle ear (otitis media with effusion), conductive hearing loss, tinnitus and otalgia [2]. These symptoms are usually unilateral and are more common in regions with a high incidence of the disease [6]. It has been postulated that the altered Eustachian tube compliance in these patients is a result of cartilage erosion by the tumor and not necessarily the destruction of the tensor veli palatinus [7]. Bilateral Eustachian tube dysfunction in nasopharyngeal cancer is rarely reported in the literature. It can occur if the tumor grows to obstruct the openings of the Eustachian tubes in the nasopharynx, especially in the exophytic or infiltrative morphological type. In that instance, the otologic presentation will initially be unilateral. In our patient, both ears were simultaneously affected after air travel. Usually, mild conductive hearing loss accompanies otitis media with effusion. However in this patient, the severe bilateral conductive hearing loss may be due to the summative effects of both the sheer bulk of the tumor in the nasopharynx and the otitic barotrauma on the Eustachian tube.

The hidden nature of the nasopharyngeal space poses diagnostic and therapeutic challenges, thus allowing significant spread of the disease before diagnosis [8]. The inclusion of nasopharyngoscopy in the clinical setting has greatly increased early diagnosis of nasopharyngeal cancer with consequently improved prognosis of the disease [9]. This was not done in our patient because nasopharyngeal cancer was not in our list of differentials. In a study by Grandawa *et al*. of 40 patients with nasopharyngeal carcinoma in north-eastern Nigeria, otologic symptoms were not noted. The clinical profile reported in these patients included cervical lymphadenopathy (72.5%), rhinorrhea (55%) and epistaxis (45%) [10]. However, a study by Iseh *et al*. of 30 patients in north-western Nigeria reported clinical presentations of deafness and otalgia in 36.3% and 30% of patients, respectively. Other clinical presentations included cervical lymphadenopathy (93.3%), epistaxis (83.3%), nasal obstruction (66.7%), palatal swelling (26.7%), cranial nerve involvement (23.3%) and visual impairment (20%) [8]. A study by Sham *et al*. of 237 Chinese patients with nasopharyngeal cancer showed that 41% of them had unilateral serous otitis media [3]. This value is quite high and may be related to the fact that nasopharyngeal cancer is seen more commonly among Asians [6]. The true incidence of this disease in Africa, however, is largely unknown: Nwaorgu *et al*. reported a steady increase in the disease occurrence over the last two decades in Nigeria [11]. Inner ear symptoms, such as vertigo, in nasopharyngeal cancer are rare [12]. In our patient, bilateral hearing impairment and tinnitus were the only presenting symptoms. Nasopharyngeal cancer is unlikely to be easily thought of as a possible diagnosis, especially when the symptoms occur after air travel. Our patient was initially treated for barotitis and only when the symptoms did not improve was she re-evaluated and a diagnosis of nasopharyngeal cancer confirmed.

Otitic barotrauma (barotitis) is a traumatic inflammation of the middle ear occurring as a result of pressure difference between the air in the middle ear and the external atmosphere, developing after ascent or, more usually, descent during air travel. It occurs because of the failure of the Eustachian tube to equilibrate middle ear and atmospheric pressure. It is quite common and presents with ear fullness, otalgia and deafness [13]. Severe cases may result in tympanic membrane perforation and even round window perforation [13]. It is an uncommon differential diagnosis of nasopharyngeal cancer [14]. The treatment of nasopharyngeal carcinoma is chemoradiation. This was the treatment administered to our patient and she has shown remarkable improvement in her clinical condition to date. The observed significant improvement in hearing thresholds in the repeat pure tone audiogram may be a result of the combined effect of both the gross tumor excision during the biopsy and chemoradiation therapy, which might have relieved the Eustachian tube obstruction.

## Conclusion

In this case report, it is suggested that isolated bilateral otologic symptoms can be the only or initial manifestation of nasopharyngeal cancer even in regions of low disease incidence. It is therefore recommended that, in cases of bilateral serous otitis media or Eustachian tube dysfunction in an adult, nasopharyngeal cancer should be considered.

## Consent

Written informed consent was obtained from the patient for the publication of this case report and any accompanying images. A copy of this consent is available for review by the Editor- in-Chief of this journal.

## Competing interests

The authors declare that they have no competing interests.

## Authors' contributions

DA was the principal investigator, performed the literature search and wrote the manuscript. FAJ assisted in preparing and proofreading the manuscript for intellectual content and gave final approval for the publication. DA and FAJ read and approved the final manuscript and take responsibility for its publication.
